# Metabolomic analysis reveals a differential adaptation process of the larval stages of *Anisakis simplex* to the host environment

**DOI:** 10.3389/fmolb.2023.1233586

**Published:** 2023-07-13

**Authors:** Iwona Polak, Robert Stryiński, Marta Majewska, Elżbieta Łopieńska-Biernat

**Affiliations:** ^1^ Department of Biochemistry, Faculty of Biology and Biotechnology, University of Warmia and Mazury in Olsztyn, Olsztyn, Poland; ^2^ Department of Human Physiology and Pathophysiology, School of Medicine, Collegium Medicum, University of Warmia and Mazury in Olsztyn, Olsztyn, Poland

**Keywords:** *Anisakis simplex*, larval development, metabolome, trehalose, fatty acids

## Abstract

**Introduction:** *Anisakis simplex* are parasitic nematodes that cause anisakiasis. The possibility of infection with this parasite is through consumption of raw or undercooked fish products. *A. simplex* infections are often misdiagnosed, especially in subclinical cases that do not present with typical symptoms such as urticaria, angioedema, and gastrointestinal allergy. The resulting allergic reactions range from rapid-onset and potentially fatal anaphylactic reactions to chronic, debilitating conditions. While there have been numerous published studies on the genomes and proteomes of *A. simplex*, less attention has been paid to the metabolomes. Metabolomics is concerned with the composition of metabolites in biological systems. Dynamic responses to endogenous and exogenous stimuli are particularly well suited for the study of holistic metabolic responses. In addition, metabolomics can be used to determine metabolic activity at different stages of development or during growth.

**Materials and methods:** In this study, we reveal for the first time the metabolomes of infectious stages (L3 and L4) of *A. simplex* using untargeted metabolomics by ultra-performance liquid chromatography-mass spectrometry.

**Results:** In the negative ionization mode (ESI-), we identified 172 different compounds, whereas in the positive ionization mode (ESI+), 186 metabolites were found. Statistical analysis showed that 60 metabolites were found in the ESI- mode with different concentration in each group, of which 21 were more enriched in the L3 larvae and 39 in the L4 stage of *A. simplex*. Comparison of the individual developmental stages in the ESI + mode also revealed a total of 60 differential metabolites, but 32 metabolites were more enriched in the L3 stage larvae, and 28 metabolites were more concentrated in the L4 stage.

**Discussion:** The metabolomics study revealed that the developmental stages of *A. simplex* differed in a number of metabolic pathways, including nicotinate and nicotinamide metabolism. In addition, molecules responsible for successful migration within their host, such as pyridoxine and prostaglandins (E1, E2, F1a) were present in the L4 stage. In contrast, metabolic pathways for amino acids, starch, and sucrose were mainly activated in the L3 stage. Our results provide new insights into the comparative metabolome profiles of two different developmental stages of *A. simplex*.

## 1 Introduction

*Anisakis simplex* is one of the most important emerging parasitic nematodes in Europe, according to the Risk Management Ranking of Foodborne Parasites, prepared for recommendations by the Food and Agriculture Organization of the United Nations (FAO) and the World Health Organization (WHO) (Stryiński et al., 2020). The disease caused by the genus *Anisakis* is called anisakiasis and frequently described (Audicana et al., 2002; Aibinu et al., 2019). The life cycle of *A. simplex* is complex and involves four larval stages parasitizing several intermediate and paratenic hosts (fish, cephalopods, and crustaceans) and the adult stage parasitizing marine mammals (seals, dolphins, and whales). The first case of human infection by a species of the Anisakidae family occurred in the 1960s, when Van Thiel of the Institute of Tropical Medicine in Leiden, the Netherlands, identified patients who suffered severe abdominal pain after eating fish (van Thiel et al., 1960). Transmission of the parasite is clearly associated with the consumption of raw or undercooked fish. In particular, Japanese sushi and sashimi, Dutch salted or smoked herring, Nordic gravlax (dry, cured salmon), Hawaiian lomi-lomi (raw salmon), German rollmops (rolled fillet of marinated/pickled herring), South American cebiche, and Spanish boquerones en vinagre (pickled anchovies) are regular routes of infection (Audicana et al., 2002; Ivanovic et al., 2015). The ingestion of viable larvae might lead to gastrointestinal symptoms (abdominal pain, nausea, vomiting, diarrhea), which may be associated with mild to severe allergic reactions, and the clinical symptoms most often are such as rhinitis, urticaria, and, in worst cases, anaphylactic shock (Aibinu et al., 2019). Although, cooking (or freezing) is expected to kill the parasites, it might not decrease its allergenicity, because *A. simplex* allergens have high heat and frost resistance; and sensitization may occur after consumption (Audicana and Kennedy, 2008). It is estimated that the incidence of the disease is 0.32 cases per 100,000 individuals/year worldwide (Orpha.net, 2022). The globalization, development of diagnostic tools and better analytical methods, have led to significantly even more anisakiasis being reported. Before 2010, over 20,000 cases of anisakiasis were reported worldwide, with the highest prevalence (over 90%) in Japan (EFSA, 2010; Baird et al., 2014), where now 7,000 cases of the disease are reported annually (Yorimitsu et al., 2013; Suzuki et al., 2021). European countries where cases of anisakiasis have been reported include among others, Spain (Herrador et al., 2019), Italy (Guardone et al., 2018; Mattiucci et al., 2018), France (Yera et al., 2018), Croatia (Mladineo et al., 2015), and Poland (Kołodziejczyk et al., 2020). The ingested L3 larvae rarely develop to L4 stage in humans and consequently die, but both of these larval stages are considered dangerous to humans (Kagei and Isogaki, 1992; Sohn et al., 2015).

The infective L3 of parasitic nematodes require specific stimuli for resumption of development and completion of shedding of the outer cuticle; thus, CO_2_ appears to be the most important stimulus for hatching or molting of *Anisakis* nematodes. In addition, temperature, pH, and pepsin are known to trigger the development of infectious L3 to adults *in vitro* (Iglesias et al., 2001, 2005). All these stimuli are thought to reflect the conditions that prevail *in vivo* when they reach the gastrointestinal tract of the definitive hosts, where they develop into adults. During this molting process, they adapt to the new environment of their hosts’ digestive tracts and also exhibit pathogenicity toward their hosts. These differences may reflect metabolic adaptations of *A. simplex* larvae to the host switch from fish (L3) to mammals (L4), i.e., adaptations to a new habitat (Baird et al., 2014; Kuhn et al., 2016).

Metabolomics is a distinct ‘omics’ method that offers a more direct assessment of physiology compared to others. It responds promptly to nutrients, stress, and disease unlike transcriptomic or proteomic approaches. Given this advantage, metabolomics has gained considerable interest in various fields such as environmental toxicology, evolutionary biology and developmental studies, medical diagnosis and treatment responses as well as drug development (Vernocchi et al., 2016). Synthetic biologists also use metabolomic flux analyses for insights into the effect of genetic modifications on metabolic pathways and products. As it pertains directly to molecular response mechanisms resulting from genetic alterations or environmental changes at the ultimate level of biological systems’ metabolism function regarding molecule abundance predictions offer better accuracy than gene expression or protein-level information provides (Liu et al., 2021). Similarly, parasitic nematode research increasingly relies on metabolomics techniques due to its immense potential applications in studying these organisms effectively. The gastrointestinal tract is a dynamic metabolic and immunologically active ecosystem, and its complete set of metabolites reflects both the enzymatic pathways of host and gut inhabitants and the complex network that connects them (Palevich et al., 2021, 2022; Whitman et al., 2021). The use of metabolomics is particularly suited to understanding nematode metabolism, including the identification of novel drug targets, differences between developmental stages, and mechanisms involved in host-parasite interactions (Jasmer et al., 2019; Molenaars et al., 2021).

A major problem with untargeted metabolomics in general is the lack of comprehensive measurements of the whole metabolome using a single technique because of its great complexity. As things stand, it is not possible to measure the entire metabolome of an organism in a single experiment. In addition to some known compounds, a large portion of the metabolome has not even been identified (“metabolic dark matter”) (da Silva et al., 2015). Metabolomics is capable of providing a static snapshot of the current metabolic state. However, in most cases, it remains unclear how this state was reached, and which metabolic pathway was active (Salzer and Witting, 2021). With respect to parasitic nematodes, the field of metabolomics faces similar challenges to untargeted metabolomics in general, as its complex nature makes it difficult to obtain complete measurements of the entire metabolome using a single approach. Nevertheless, the use of metabolomics in the study of parasitic nematodes is a promising area that could provide important insights into their biology and pathogenesis.

Such studies on the metabolome of nematodes from the family Anisakidae are lacking. Therefore, to fill the described gap, it was decided to analyze and characterize the metabolome of *A. simplex*, a parasite of great public health importance. In the central part of this work, we focused on the identification and characterization of the metabolites of *A. simplex* in two developmental stages to increase our knowledge of the biology of this organism and to find a way to understand how this parasite was able to adapt to different host environments by developing a unique metabolism.

## 2 Materials and methods

### 2.1 Parasites and *in vitro* culture

The study was performed on the L3 and L4 larval stages of *Anisakis simplex* Nematodes were collected from the Biobank platform implemented for the PARASITE project (www.parasite-project.eu) at Institute of Marine Research, Spanish National Research Council (IIM-CSIC), Vigo, Spain. The larval stages of *A. simplex* were isolated: L3 from hake (*Merluccius*) and L4 from striped dolphin (*Stenella coeruleoalba*). All used larvae were taxonomically identified using conventional PCR to amplify the ITS region and *Cox2* gene as described before by Levsen et al. (2018). Eight samples of L3 larvae (24 larvae in total) and four samples of L4 larvae (8 larvae in total) were preserved in −80°C until the time of next step of the analysis.

### 2.2 Samples preparation

Samples were thawed and add with 800 μL of 80% methanol. Then all samples were extracted at 4°C with ultrasound for 30 min, kept at −40°C for 1 h. After that, samples were vortexed for 30 s, and centrifuged at 12,000 rpm and 4°C for 15 min. Finally, 200 μL of supernatant and 5 μL of DL-o-chlorophenylalanine (140 μg/ml) was transferred to vial for LC-MS/MS analysis. Quality control (QC) samples were used to evaluate the methodology. The same amount of extract was obtained from each sample and mixed as QC samples. The QC samples (3 in total) were prepared using the same sample preparation procedure.

### 2.3 UPLC-TOF-MS/MS

The separation of compounds was performed by ultra-performance liquid chromatography coupled with tandem mass spectrometry using Ultimate 3000LC combined with Q Exactive MS (Thermo Fisher Scientific, Waltham, MA, United States) in both polarities (ESI −/+). The LC system was comprised of a Hyper gold C18 column (100 × 2.1 mm 1.9 μm) (Thermo Fisher Scientific, Waltham, MA, United States). The mobile phase was composed of solvent A (0.1% formic acid, 5% acetonitrile, HPLC-grade water) and solvent B (0.1% formic acid, acetonitrile) with a gradient elution (0–1.5 min, 0%–20% B; 1.5–9.5 min, 20%–100% B; 9.5–14.5 min, 100% B; 14.5–14.6 min, 100%–0% B; 14.6–18.0 min, 0% B). The flow rate of the mobile phase was 0.3 ml/min. The column temperature was maintained at 40°C, and the sample manager temperature was set at 4°C. Mass spectrometry parameters in ESI+ and ESI- mode are listed as follows: the sheath gas flow rate was set to 45 a. u. (arbitrary units), aux gas flow rate to 15 a. u., and sweep gas flow rate to 1 a. u., capillary temperature was 350°C, and probe heater temperature was 300°C. Electrospray ionization source was set to 3.0 kV in ESI+ and 3.2 kV in ESI-.

### 2.4 Data processing and analysis

The raw LC-MS/MS data were acquired and aligned using the Compound Discover (v. 3.0, Thermo Fisher Scientific, Waltham, MA, United States) based on the *m/z* value and the retention time (RT) of the ion signals. The spectral data were normalized, and auto scaled before statistical analysis. The data was introduced into the SIMCA-P software (version 14.1, Santorius, Goettingen, Germany) for multivariate analysis. A Principal Components Analysis (PCA) was first used as an unsupervised method for data visualization and outlier identification (Yeshi et al., 2020). Supervised regression modeling was performed on the data set by use of Partial Least Squares Discriminant Analysis (PLS-DA). The quality of the models was evaluated with the relevant *R* ^*2*^ and *Q* ^*2*^ *.* The importance of each ion in the PLS-DA was evaluated by variable importance in the projection (VIP) score (Tambellini et al., 2013; Gao et al., 2016). The VIP score positively reflects the metabolite’s influence on the classification, and metabolites with VIP >1 were considered important in the study.

The chemical structures and IDs for metabolites were identified according to Human Metabolome Database (Wishart et al., 2012), KEGG database (Kanehisa and Goto, 2000; Kanehisa et al., 2016), PubChem Compound ID database (Kim et al., 2022) and ChemSpider databse (https://www.chemspider.com/Default.aspx). When necessary, further identification was performed through comparisons of the retention times and MS/MS fragmentation patterns in other databases: Metabolite and Chemical Entity Database (METLIN) (Smith et al., 2005) or MassBank (https://massbank.eu/MassBank/) (Salek et al., 2013).

The metabolites showing different concentrations among two given groups (DMs) were filtered and confirmed by combining the results of the multivariate analysis (VIP values >1.0) and the results of univariate analyses: *t*-test (*p*-value ≤0.05) and fold-change (FC) method (-1.5 ⩽ Log_2_FC ⩽ 1.5) for both ESI modes. Data were visualized on volcano plots.

In multivariate analyses, the hierarchical clustering analysis (HCA) with Euclidean measured distance, and the average clustering algorithm was used to visualize the differences in the concentration of each statistically significant metabolites between groups in two different ESI modes. Subsequently, the metabolites assignment to the compounds groups and pathway enrichment analysis was performed using Metaboanalyst 5.0 (Xia et al., 2009; Pang et al., 2021). The enrichment overview was based on the KEGG database with *Caenorhabditis elegans* as a reference (Kanehisa and Goto, 2000; Kanehisa et al., 2016).

Furthermore, the distribution of common and unique metabolites identified in the two ESI modes was analyzed and visualized using the Venn Diagrams tool (https://bioinformatics.psb.ugent.be/webtools/Venn/).

## 3 Results

### 3.1 Metabolites identification and statistical analysis

The QC samples were used to demonstrate the stability of the LC-MS system. The QC samples run in positive and negative mode at regular intervals throughout the entire sequence. The ion features of the QC samples were used to calculate the relative standard deviation (RSD). The %RSD distribution for negative and positive modes is presented in Supplementary Figures S1A, B, respectively; an overwhelming majority of the RSD is less than 30%. The base peak intensity (BPI) chromatograms of the QC samples in ESI−/+ modes are presented in Supplementary Figures S1C, D. The normalization after alignment was also performed and the line plot (Supplementary Figures S1E, F) was used to evaluate the methodology. The *X* axis indicates the number of samples, the *Y* axis indicates the 95% confidence interval. The line plot demonstrated that the system is relatively stable during sample analysis. Due to all of that, the analysis procedure was robust and could be used for subsequent sample analysis.

**FIGURE 1:**
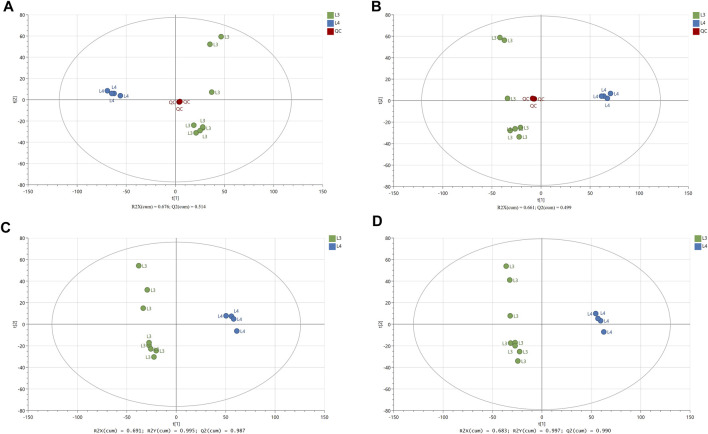
The results of multivariate data analysis. The score scatter plots of PCA model **(A, B)** comparing LC-MS/MS metabolomic profiles for the L3 and L4 developmental stages of *A. simplex* in negative and positive ionization mode (ESI−/+), respectively, and QC group. The score scatter plots of PLS-DA model **(C, D)** comparing LC-MS/MS metabolomic profiles for the L3 and L4 developmental stages of *A. simplex* in negative and positive ionization modes (ESI−/+), respectively. The *R* ^*2*^ and *Q* ^*2*^ values are indicated in the figures.

To investigate the global metabolism variations, the PCA analysis was used to analyze all observations acquired in both ion modes. As show in PCA plot (Figures 1A, B), the QC samples were successfully separated from the tested samples and clustered together (QC, L3 and L4 developmental stages of *A. simplex*). The parameters *R* ^*2*^ and *Q* ^*2*^ confirmed the validity of the PCA model as follows: ESI- mode, *R* ^*2*^ *X* = 0.676, *Q* ^*2*^ = 0.514; ESI + mode, *R* ^*2*^ *X* = 0.661*, Q* ^*2*^ = 0.499 (Figures 1A, B). To eliminate any non-specific effects of the operative technique and confirm the presence of DMs, the PLS-DA was performed. The PLS-DA score scatter plots showed that there was significant separation between the L3 developmental stage group and the L4 developmental stage group in the ESI- and ESI + modes (Figures 1C, D). The values of the cumulative *R* ^*2*^ and *Q* ^*2*^ parameters confirmed the validity of the PLS-DA model: ESI- mode, *R* ^*2*^ *X* = 0.691, *R* ^*2*^ *Y* = 0.995, and *Q* ^*2*^ = 0.987; ESI + mode, *R* ^*2*^ *X* = 0.683, *R* ^*2*^ *Y* = 0.997, and *Q* ^*2*^ = 0.990 (Figures 1C, D). Additionally, the CV-ANOVA analysis assessing the reliability of the PLS-DA model was performed (Supplementary Figure S2). The *p*-value obtained for ESI- and ESI + modes was less than 0.05: 7.3415 × 10^−6^, and 3.998 × 10^−6^, respectively. The CV-score plots of the samples in ESI- and ESI + modes showed, as well, clear separation of the two analyzed groups with F factor = 77.64 and 91.61 for negative and positive ionization modes, respectively (Supplementary Figures S2A, B). According to the permutation test results, the PLS-DA model was proved to have good robustness without over fitting (Supplementary Figures S2C, D).

**FIGURE 2:**
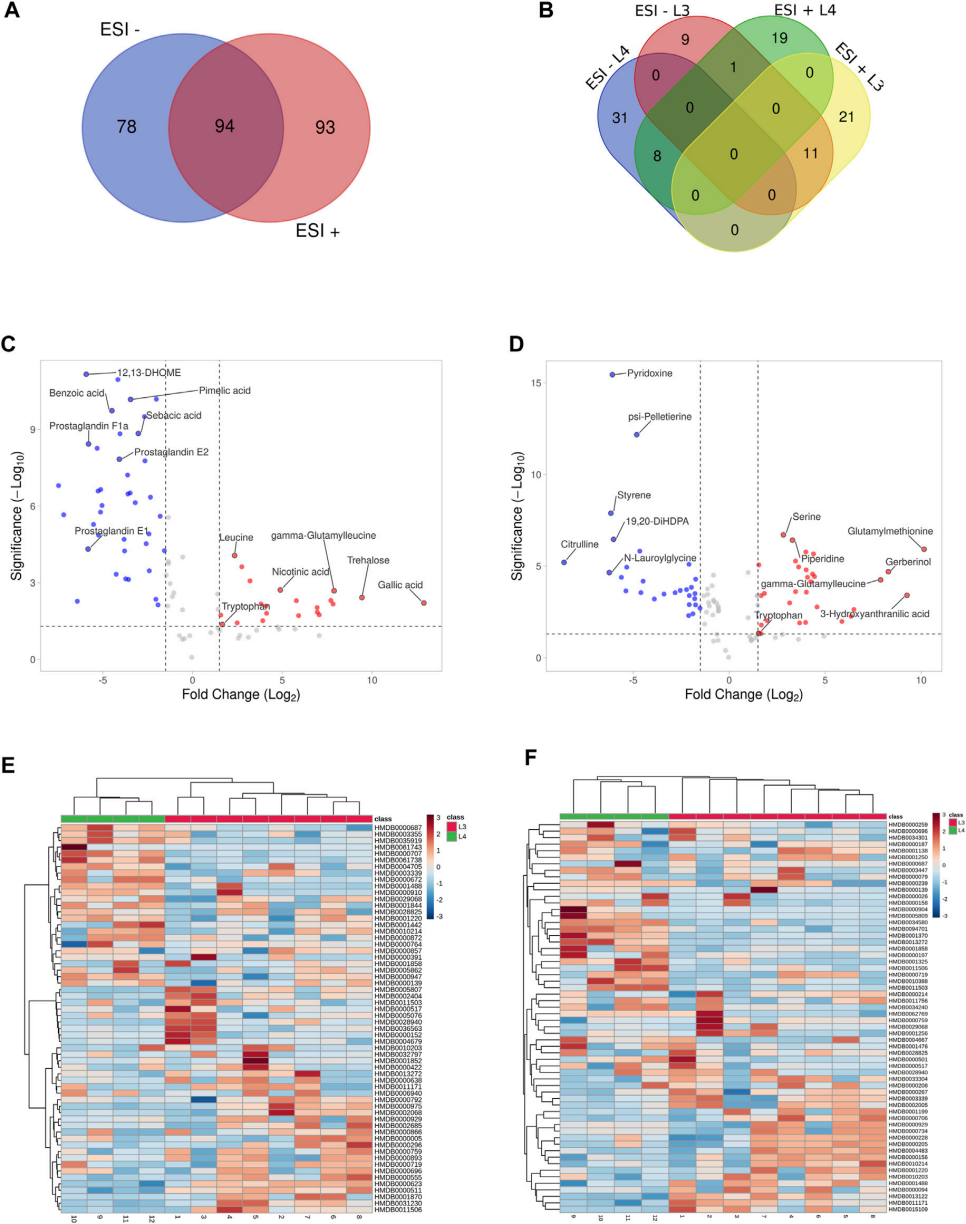
Visualization of statistically significant data results. Distribution of common and unique metabolites identified in the study between negative and positives ionization modes (ESI−/+) **(A)**. The metabolites IDs are listed in Supplementary Table S7. Distribution of common and unique metabolites for each of *A. simplex* developmental stage (L3 and L4) identified in the study in negative and positives ionization modes and (ESI−/+) **(B)**. The metabolites IDs are listed in Supplementary Table S10. Volcano plots of the untargeted metabolomics analysis in negative and positive ionization modes (ESI−/+), respectively **(C, D)**, of two developmental stages of *A. simplex*. The metabolites showing different concentrations among the given groups (DMs) were filtered and confirmed by combining the results of the multivariate analysis (VIP values >1.0) and the results of univariate analyses (-1.5 ⩽ Log_2_FC ⩽ 1.5 in normalized ratios of L3 vs L4, *p*-value ≤0.05) for both ESI modes. Metabolites more enriched in the L3 stage are coloured in red, and in the L4 stage in blue. The metabolites IDs with calculated *p*-values, FC and VIP are listed in Supplementary Tables S8, 9. Hierarchical clustering analysis of the two sample groups (L3 and L4 stages of *A. simplex*) in negative (ESI-) and positive (ESI+) ionization modes, respectively **(E, F)**. The HCA shows discrimination between the samples groups and differential abundances of DMs in ESI- **(E)** and ESI+ **(F)**. The scale bars represent the normalized intensity of metabolites, where blue indicates a decrease/low and red an increase/high.

As a result of LC-MS/MS analysis, we identified a total of 3603 and 3877 compounds (Supplementary Tables S1, 2) in ESI- and ESI+, respectively. The peak intensities after normalization against QC samples (Supplementary Tables S3, 4) were further processed and the chemical structures and IDs for metabolites were identified (Supplementary Tables S5, 6). In the ESI- we identified 172 different compounds (Supplementary Table S5), when in ESI +, 186 metabolites were found (Supplementary Table S6). It was checked whether the identified compounds overlap and occur similarly in both used polarization modes (Figure 2A). Analysis showed that 94 metabolites are common for both ESI modes (Supplementary Table S6).

These data were further processed, and several filters were next applied to obtain the final list of DMs among the given groups: a) -1.5 ⩽ Log_2_FC ⩽ 1.5 in normalized ratios (L3 vs L4), b) *t*-test (*p*-value ≤0.05), c) VIP >1.0. Afterwards, the significantly changed metabolites between the two groups were filtered out and listed in Supplementary Tables S8, 9. Statistical analysis showed that in ESI- mode was 60 DMs, of which 21 were more detected in the L3 larvae and 39 in the L4 stage of *A. simplex* (Supplementary Table S8). Comparison of the individual developmental stages in ESI + mode revealed, as well, a total of 60 DMs, however with 32 metabolites more enriched in the L3 stage larvae and 28 metabolites more enriched in the L4 stage (Supplementary Table S9). The distribution of common and unique metabolites identified in the two ESI modes specific for each developmental stage was also analyzed and visualized with the use of Venn diagram (Figure 2B, Supplementary Table S10). It was found that there are 11 common metabolites between the ESI −/+ modes for the L3 stage (l-arginine, tryptophan, threonylphenylalanine, homoserine, leucyl-tryptophan, glutamylmethionine, gamma-glutamylleucine, glutamic acid, l-methionine, leucine, and glycylleucine), and eight for the L4 stage (13-HOTE, LysoPE (18:1 (9Z)/0:0), prostaglandin E2, N-lauroylglycine, glyceric acid, LysoPE (16:0/0:0), p-cresol, and 19,20-DiHDPA) (Figure 2B, Supplementary Table S10). The volcano plot representations of DMs are shown in Figures 2C, D. Among the DMs more enriched for L3 stage larvae, found in ESI-, we identified gallic acid and trehalose, as those with the highest fold change. Moreover, in the group of metabolites more enriched in L3 we found, e.g., nicotinic acid, leucine, tryptophan or gamma-glutamylleucine (Figure 2C; Table 1). In the studied polarization (ESI-), such compounds as pimelic acid, sebacic acid, benzoic acid, or prostaglandins E1, E2, F1a were found in increased concentration in L4 larvae compared to L3 (Figure 2C; Table 1). The analysis of the *A. simplex* metabolome in ESI + mode showed higher concentration in L3 compared to L4 of such metabolites as: glutamylmethionine, serine, piperidine or 3-hydroxyanthranilic acid (Figure 2D; Table 2). Moreover, in ESI + mode, increased amounts of e.g., pyridoxine, psi-pelletierine, styrene, citrulline or N-laurolylglicine were observed in L4 larvae in relation to the L3 stage (Figure 2D; Table 2). The HCA was used to visualize the differences in the concentration of each statistically significant metabolite between L3 and L4 developmental stages of *A. simplex* in two different ESI modes (Figures 2E,F). An overview on the DMs concentrations from all samples can be observed and clear clustering and grouping trend between L3 and L4 developmental stages of *A. simplex* is showed.

**TABLE 1:** Metabolites of developmental stages of *A. simplex* (L3 and L4) identified in negative ionization mode (ESI-).

Hmdb ID	Compound name	Log_2_(FC)	*p*-value	Stage with increased concentration
HMDB0005807	Gallic acid	12.89	0.006171	L3
HMDB0000005	2-Ketobutyric acid	2.49	0.037232	L3
HMDB0011171	gamma-Glutamylleucine	7.89	0.002065	L3
HMDB0003355	5-Aminopentanoic acid	2.75	0.000237	L3
HMDB0000687	Leucine	2.34	8.59E-05	L3
HMDB0003339	Glutamic acid	3.82	0.006789	L3
HMDB0000696	l-Methionine	4.13	0.015656	L3
HMDB0028825	Glutamylmethionine	4.09	0.008103	L3
HMDB0029068	Threonylphenylalanine	3.91	0.030709	L3
HMDB0001488	Nicotinic acid	4.89	0.001914	L3
HMDB0000975	Trehalose	9.44	0.003788	L3
HMDB0028940	Leucyl-Tryptophan	7.83	0.006797	L3
HMDB0000719	Homoserine	5.91	0.019309	L3
HMDB0002068	Erucic acid	1.57	0.018429	L3
HMDB0000929	Tryptophan	1.66	0.042818	L3
HMDB0000152	Gentisic acid	6.95	0.009357	L3
HMDB0000517	l-Arginine	7.06	0.017952	L3
HMDB0000296	Uridine	5.79	0.005042	L3
HMDB0000759	Glycylleucine	6.95	0.01415	L3
HMDB0000866	N-Acetyl-l-tyrosine	3.20	0.000844	L3
HMDB0002404	Alpha-Hydroxyhippuric acid	7.71	0.004941	L3
HMDB0000623	Dodecanedioic acid	-2.02	6.54E-11	L4
HMDB0036563	Valerenolic acid	-3.48	3.07E-07	L4
HMDB0000947	Undecanoic acid	-4.04	1.48E-09	L4
HMDB0001870	Benzoic acid	-4.49	1.85E-10	L4
HMDB0001220	Prostaglandin E2	-4.08	1.46E-08	L4
HMDB0000139	Glyceric acid	-3.62	6.09E-08	L4
HMDB0004679	8-HETE	-3.19	7.36E-07	L4
HMDB0000857	Pimelic acid	-3.45	6.7E-11	L4
HMDB0000792	Sebacic acid	-3.02	1.44E-09	L4
HMDB0002685	Prostaglandin F1a	-5.80	3.72E-09	L4
HMDB0000872	Tetradecanedioic acid	-4.16	1.11E-11	L4
HMDB0000511	Capric acid	-2.65	1.69E-08	L4
HMDB0000910	Tridecanoic acid	-1.80	2.49E-06	L4
HMDB0000555	3-Methyladipic acid	-5.32	5.45E-09	L4
HMDB0001858	p-Cresol	-5.12	2.25E-07	L4
HMDB0004705	12,13-DHOME	-5.92	6.92E-12	L4
HMDB0035919	Corchorifatty acid F	-5.27	2.57E-07	L4
HMDB0031230	2-Ethylhexanoic acid	-2.68	3.15E-10	L4
HMDB0061743	Perfluoroundecanoic acid	-7.47	1.57E-07	L4
HMDB0000707	4-Hydroxyphenylpyruvic acid	-5.13	1.72E-06	L4
HMDB0000672	Hexadecanedioic acid	-5.04	9.47E-07	L4
HMDB0005076	13,14-Dihydro PGF-1a	-3.81	1.99E-05	L4
HMDB0000764	Hydrocinnamic acid	-7.18	2.19E-06	L4
HMDB0013272	N-Lauroylglycine	-5.52	5.22E-06	L4
HMDB0000893	Suberic acid	-2.34	4.47E-07	L4
HMDB0061738	Perfluorodecanoic acid	-5.24	1.37E-05	L4
HMDB0000638	Dodecanoic acid	-1.57	5.55E-05	L4
HMDB0001852	Retinoic acid	-3.79	5.68E-05	L4
HMDB0001442	Prostaglandin E1	-5.82	4.8E-05	L4
HMDB0010203	13-HOTE	-2.43	1.23E-05	L4
HMDB0000391	7-Ketodeoxycholic acid	-3.60	3.35E-07	L4
HMDB0010214	19,20-DiHDPA	-3.57	0.000744	L4
HMDB0032797	Jasmonic acid	-4.26	0.000464	L4
HMDB0001844	Methylsuccinic acid	-2.58	2.97E-05	L4
HMDB0011503	LysoPE (16:0/0:0)	-2.41	0.00034	L4
HMDB0006940	9(S)-HPODE	-3.73	0.000702	L4
HMDB0000422	2-Methylglutaric acid	-2.04	0.004432	L4
HMDB0011506	LysoPE (18:1 (9Z)/0:0)	-1.92	0.007341	L4
HMDB0005862	2-Methylguanosine	-6.42	0.005289	L4

**TABLE 2:** Metabolites of developmental stages of *A. simplex* (L3 and L4) identified in positive ionization mode (ESI+).

Hmdb ID	Compound name	Log_2_(FC)	*p*-value	Stage with increased concentration
HMDB0094701	N-Acetylproline	1.83	0.00030629	L3
HMDB0000687	Leucine	1.54	0.00000855	L3
HMDB0000696	l-Methionine	3.15	0.00101515	L3
HMDB0033304	Gerberinol	8.29	0.00002015	L3
HMDB0034301	Piperidine	3.30	0.00000038	L3
HMDB0003339	Glutamic acid	4.44	0.00003730	L3
HMDB0000517	l-Arginine	6.49	0.00234735	L3
HMDB0000267	Pyroglutamic acid	3.99	0.00000169	L3
HMDB0000156	Malic acid	4.08	0.00004064	L3
HMDB0000719	Homoserine	3.45	0.00000530	L3
HMDB0011171	gamma-Glutamylleucine	7.88	0.00005651	L3
HMDB0001476	3-Hydroxyanthranilic acid	9.26	0.00039118	L3
HMDB0028825	Glutamylmethionine	10.15	0.00000120	L3
HMDB0001138	N-Acetyl-l-glutamic acid	3.46	0.00024656	L3
HMDB0000929	Tryptophan	1.54	0.04665395	L3
HMDB0000734	Indoleacrylic acid	1.54	0.04653269	L3
HMDB0028940	Leucyl-Tryptophan	3.98	0.01189554	L3
HMDB0000187	Serine	2.82	0.00000020	L3
HMDB0001325	N6,N6,N6-Trimethyl-l-lysine	1.65	0.04833034	L3
HMDB0000228	Phenol	4.01	0.00001597	L3
HMDB0001250	N-Acetylarylamine	3.62	0.00001107	L3
HMDB0001199	N2-Succinyl-l-ornithine	4.57	0.00169112	L3
HMDB0000026	Ureidopropionic acid	4.39	0.00000215	L3
HMDB0000205	Phenylpyruvic acid	4.34	0.00002708	L3
HMDB0000759	Glycylleucine	6.35	0.00550864	L3
HMDB0011756	N-Acetylleucine	3.67	0.01260170	L3
HMDB0013122	LysoPC(P-18:0/0:0)	1.66	0.00040691	L3
HMDB0000158	l-Tyrosine	4.24	0.00007212	L3
HMDB0000214	Ornithine	1.67	0.01629332	L3
HMDB0003447	Tryptophol	1.98	0.00949214	L3
HMDB0029068	Threonylphenylalanine	5.87	0.01050157	L3
HMDB0000706	Aspartylphenylalanine	4.02	0.00026174	L3
HMDB0062769	Epsilon-caprolactam	-1.75	0.00005150	L4
HMDB0034580	psi-Pelletierine	-4.81	0.00000000	L4
HMDB0000239	Pyridoxine	-6.08	0.00000000	L4
HMDB0010214	19,20-DiHDPA	-6.02	0.00000035	L4
HMDB0034240	Styrene	-6.16	0.00000001	L4
HMDB0001858	p-Cresol	-4.65	0.00000152	L4
HMDB0002005	Methionine sulfoxide	-2.09	0.00000794	L4
HMDB0000904	Citrulline	-8.60	0.00000631	L4
HMDB0001370	Diaminopimelic acid	-5.33	0.00001148	L4
HMDB0013272	N-Lauroylglycine	-6.24	0.00002255	L4
HMDB0001220	Prostaglandin E2	-2.08	0.00126021	L4
HMDB0010203	13-HOTE	-2.81	0.00028251	L4
HMDB0001256	Spermine	-1.78	0.00031453	L4
HMDB0005809	Eugenol	-5.61	0.00004089	L4
HMDB0004667	13-HODE	-4.48	0.00006656	L4
HMDB0000197	Indoleacetic acid	-2.55	0.00021292	L4
HMDB0004483	Estrone glucuronide	-5.38	0.00022515	L4
HMDB0010388	LysoPC(18:3 (9Z,12Z,15Z)/0:0)	-4.60	0.00028059	L4
HMDB0000139	Glyceric acid	-1.52	0.00197458	L4
HMDB0000259	Serotonin	-3.90	0.00062622	L4
HMDB0000079	Dihydrothymine	-3.39	0.00033464	L4
HMDB0001488	Nicotinic acid	-1.97	0.00014390	L4
HMDB0000094	Citric acid	-2.22	0.00019260	L4
HMDB0000501	7-Ketocholesterol	-1.89	0.00398334	L4
HMDB0011503	LysoPE (16:0/0:0)	-1.80	0.00059878	L4
HMDB0000208	Oxoglutaric acid	-2.08	0.00040543	L4
HMDB0011506	LysoPE (18:1 (9Z)/0:0)	-2.11	0.00499579	L4
HMDB0015109	Edetic Acid	-1.75	0.00130870	L4

### 3.2 Metabolite classes and enriched pathways

**FIGURE 3:**
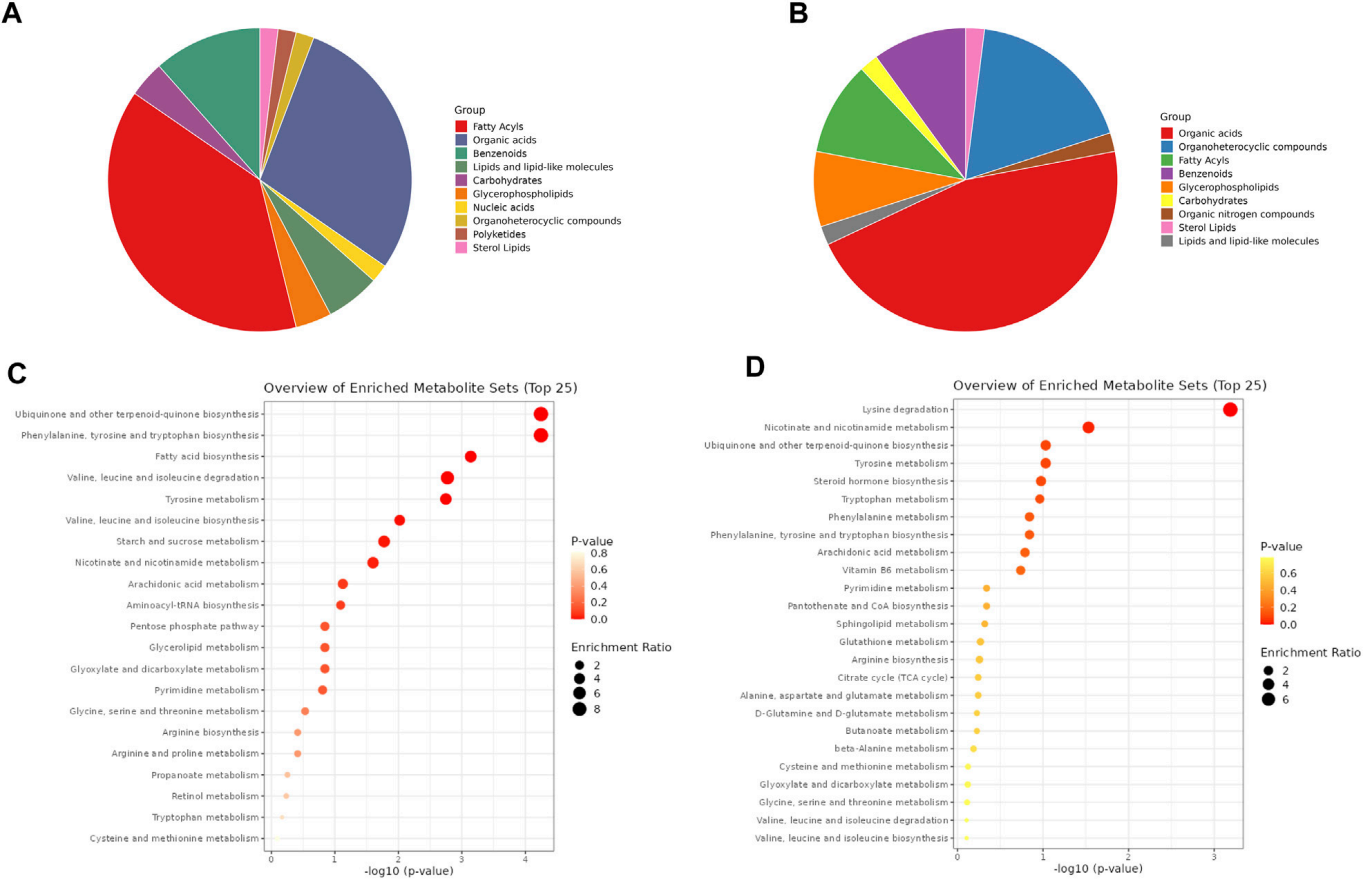
Metabolite classes and enriched pathways. Distribution of DMs by different metabolite classes identified in negative and positive ionization modes, respectively **(A, B)**. The metabolites names assigned to different classes are listed in Supplementary Tables S11, 12. Dot plots of pathway enrichment analysis of DMs identified in negative and positive ionization modes, respectively **(C, D)**. The scale bars represent *p*-value and enrichment ratio. The pathways names with calculated *p*-value are listed in Supplementary Tables S13, 14.

The DMs identified in ESI- and ESI+ (60 in each mode) were assigned to different metabolite classes/groups (Supplementary Tables S11, 12). The highest number of metabolites identified in ESI- mode were categorized into the fatty acyls (e.g., prostaglandin E2, prostaglandin E1, prostaglandin F1a, 8-HETE, 13-HOTE), organic acids (e.g., sebacic acid, pimelic acid, suberic acid, methylsuccinic acid), and benzenoids (p-cresol, benzoic acid, alpha-hydroxyhippuric acid) (Figure 3A, Supplementary Table S11). Of 60 DMs identified in ESI + mode, 23 of them were organic acids (e.g., ureidopropionic acid, citric acid, l-malic acid), nine were organoheterocyclic compounds (dihydrothymine, indoleacetic acid, pyridoxine, serotonin, pyroglutamic acid, indoleacrylic acid, nicotinic acid, tryptophanol, piperidine), 10 were fatty acyls and benzenoids, and four were glycerophospholipids (e.g., LysoPC(18:3 (9Z,12Z, 15Z)); LysoPE (16:0/0:0); LysoPE (18:1 (9Z)/0:0); LysoPC(P-18:0)) (Figure 3B, Supplementary Table S12).

Based on the DMs identified in both developmental stages of *A. simplex* in ESI−/+ ionization modes, the metabolic pathways were mapped, and enrichment analysis was performed (*p*-value ≤0.05) (Figures 3C, D; Supplementary Tables S13, 14). In negative ionization mode (ESI-) the highest number of identified DMs were products of ubiquinone and other terpenoid-quinone biosynthesis (KEGG 00130), followed by phenylalanine, tyrosine and tryptophan biosynthesis (KEGG 00400), fatty acid biosynthesis (KEGG 00061), valine, leucine and isoleucine biosynthesis and degradation (KEGG 00290, 00280), tyrosine metabolism (KEGG 00350), starch and sucrose metabolism (KEGG 00500), and nicotinate and nicotinamide metabolism (KEGG 00760) (Kanehisa and Goto, 2000; Kanehisa et al., 2016). Mapping and enrichment analysis of pathways for identified DMs in positive ionization mode (ESI+) against known pathways in KEGG database, showed that highest number of DMs were products of lysine degradation (KEGG 00310), and nicotinate and nicotinamide metabolism (KEGG 00760) (Kanehisa and Goto, 2000; Kanehisa et al., 2016).

## 4 Discussion

One of the most recent additions to the *Caenorhabditis elegans* toolbox is metabolomics and lipidomics, which allow new and deeper investigations of nematode metabolism. Combining a genetically defensible model organism such as *C. elegans* with the functional evaluation of metabolomics and/or lipidomics holds great promise for expanding our knowledge of metabolism and metabolic regulation (Salzer and Witting, 2021). While there are numerous published studies on the genomes (Mattiucci et al., 2016; Coghlan et al., 2019; Łopieńska-Biernat et al., 2019), transcriptomes (Cavallero et al., 2018; Kim et al., 2018; Llorens et al., 2018; Łopieńska-Biernat et al., 2018; Cavallero et al., 2020), and proteomes (Stryiński et al., 2019; Stryiński et al., 2022; Polak et al., 2020; Mierzejewski et al., 2021; Kochanowski et al., 2022) of *A. simplex*, less attention has been paid to its metabolome. Metabolic changes between L3 and L4 larval stages of *A. simplex* have been studied previously, however in a narrow range, e.g., of one metabolic pathway (Łopieńska-Biernat et al., 2008; Łopieńska-Biernat et al., 2018; Łopieńska-Biernat et al., 2019), and almost nothing is known about the complement of an intermediate or end product of metabolism in *Anisakis* nematodes, especially in the stages found in humans (L3 and L4).

In the first stage of infection the larvae are invading the host intestinal tissues and establishing in the gastrointestinal tract of the host. Such tissue migration requires larvae to adapt to constantly changing external environments, in terms of temperature, oxygen supply, redox potential and host immune reactions (Huang et al., 2020; Yeshi et al., 2020). In this metabolomic study, we observed substantial differences in the composition and abundance of trehalose, nicotinic acid, prostaglandins, and acyl acids, likely associated with key biological functions, including energy metabolism and nicotinate and nicotinamide metabolism during the parasite’s growth and development. Alterations in metabolomic profiles are likely reflected in the adaptation of *A. simplex* nematode to changing environments within the host (poikilotherm and homeotherm organisms). Nevertheless, comparative analyses of levels of metabolites in the L3 and L4 of these two larval stages led to the identification of groups of molecules with putative roles in mechanisms of parasite pathogenicity.

Our results revealed that specific to L3 are nicotinic acid and branched-chain and aromatic amino acids (BCAA). The recent metabolomics studies showed that BCAA are positively related to longevity common to Dauer in *C. elegans* and *Haemonhus contortus* during mitochondrial biogenesis in ensheathment eggs (Palevich et al., 2022). In this study, BCAA were only identified in L3 stage, which like a Dauer stage, uses endogenous energy sources. Starvation-induced stress may invoke a series of regulations of metabolic pathways (Fuchs et al., 2010; Łopieńska-Biernat et al., 2019). In this study, some pathways, including fatty acid metabolism, nicotinate/nicotinamide metabolism, terpenoid backbone biosynthesis were only identified in L3 stage.

Fatty acids serve as energy storage and structural components in biomembranes. The main chemical composition of the cuticular lipids in *A. simplex* from Atlantic cod (*Godus morhua*) was recognized as three fatty acids, 15 triacylglycerols, five sterols and 12 sphingolipids (Mika et al., 2010) what is consisted with our current results. Moreover, we have identified fatty acyls and glycerophospholipids in L4 stage larvae.

Terpenoids are the precursor in the production of steroids and sterols. Therefore, the constant activity of the terpenoid backbone biosynthesis pathway is important for the regulation of steroids metabolism, as they regulate a variety of developmental and biological processes (molting, larval development, and innate immunity) (Hernández-Bello et al., 2011). The ubiquinone/terpenoid quinone biosynthesis (energy metabolism) involved in biosynthesis of other secondary metabolites, and metabolism of cofactors (nicotinate/nicotinamide metabolism) and vitamins is specific to two larval stages. Based on current results and previous studies, energy metabolism of *A. simplex* is based on carbohydrates metabolism, where L3 larvae is metabolizing trehalose, and L4 larvae glycogen (Łopieńska-Biernat et al., 2006, Łopieńska-Biernat et al., 2007; Łopieńska-Biernat et al., 2008; Łopieńska-Biernat et al., 2018; Łopieńska-Biernat et al., 2019). However, based on the current results we can suppose that L4 larvae energy metabolism is also related to fatty acids (Ma et al., 2019). This could be an adaptation due to different host environments and usage of energy sources.

In addition, we have identified significant amounts of the prostaglandin PGE2 in L4 larvae of *A. simplex*, which helps larvae successfully migrate into their host and acts as a mediator of host immunity. These molecules mediate and have an immunosuppressive effect on T cells (Tebbey and Buttke, 1990). Genes of the prostaglandin synthesis cascade (Fitzpatrick, 2004), including cyclooxygenase (WormBase ParaSite ASIM_0001219401), alpha-tubulin N-acetyltransferaseare (WormBase ParaSite ASIM_0001506401), and phospholipase A2 (WormBase ParaSite ASIM_0001246201) are present in the genome of the nematode studied (Coghlan et al., 2019), demonstrating that *A. simplex* larvae can synthesize prostaglandins. Moreover, studies on *Trichuris suis* may provide insight into the mechanisms by which the worm suppresses inflammatory host responses, with one active component identified as prostaglandin PGE2 (Laan et al., 2017).

Lipidome analysis of *Schistosoma mansoni* has detected eicosanoids, signaling molecules made by the enzymatic or non-enzymatic oxidation of polyunsaturated fatty acids, that promote a Th2 response (Shepherd et al., 2015; Whitman et al., 2021). Metabolites analysis in this study specific to L4 stage also revealed eicosanoids. However, studies show that some helminth-derived small molecules have distinct immunomodulatory components, suggesting that further studies are needed at this host-pathogen interface (Shepherd et al., 2015; Whitman et al., 2021).

Our metabolomics study showed that *A. simplex* induced metabolic changes in a variety of metabolic pathways in both larval stages, including amino acid metabolism, phospholipid metabolism, energy metabolism, nicotinate/nicotinamide metabolism and ubiquinone/terpenoid quinone biosynthesis. In addition, several stage-specific metabolites were identified, providing potential clues for understanding the molecular mechanisms of this parasite biology: its pathogenicity and adaptation to the host environment.

By undertaking the first small-scale analysis of the metabolome molecules present in the L3 and L4 of *A. simplex* that are putatively involved in the host environment, we provide here a ready-to-use molecular groundwork for in-depth studies of the biological pathways specifically involved in parasite growth and development. Further studies under experimental, controlled *in vitro* conditions, as well as between other developmental stages of the life cycle, are needed to reliably assess the role of these molecules in the pathogenesis of anisakiasis. A snapshot of the metabolic status in specific age of the larvae and/or tissues of larval stages of *A. simplex* could provide new information on the expression of target metabolites potentially involved in host invasion. Furthermore, 13C flux experiments can provide adequate information on the route of formation of key metabolites.

Gastrointestinal nematodes strategy mediated through the carbohydrates and lipid metabolites and employed for the manipulation of the host immune response towards successful propagation and parasitism, opens a wide perspective that should be compounded by the contemporary use of multi-omics approaches.

## Data Availability

The raw data generated for this study can be found in the EMBL-EBI MetaboLights database (www.ebi.ac.uk/metabolights/MTBLS6458) with the identifier MTBLS6458.
