# Supplementary material for: Metabolomic analysis reveals a differential adaptation process of the larval stages of Anisakis simplex to the host environment
Source: Front Mol Biosci. 2023 Jul 13;10:1233586. doi: 10.3389/fmolb.2023.1233586 (PMC10373882; doi:10.3389/fmolb.2023.1233586)
Supplement: Supplementary file 2 [file Table1.DOCX]

Supplementary Material

# Supplementary Figures


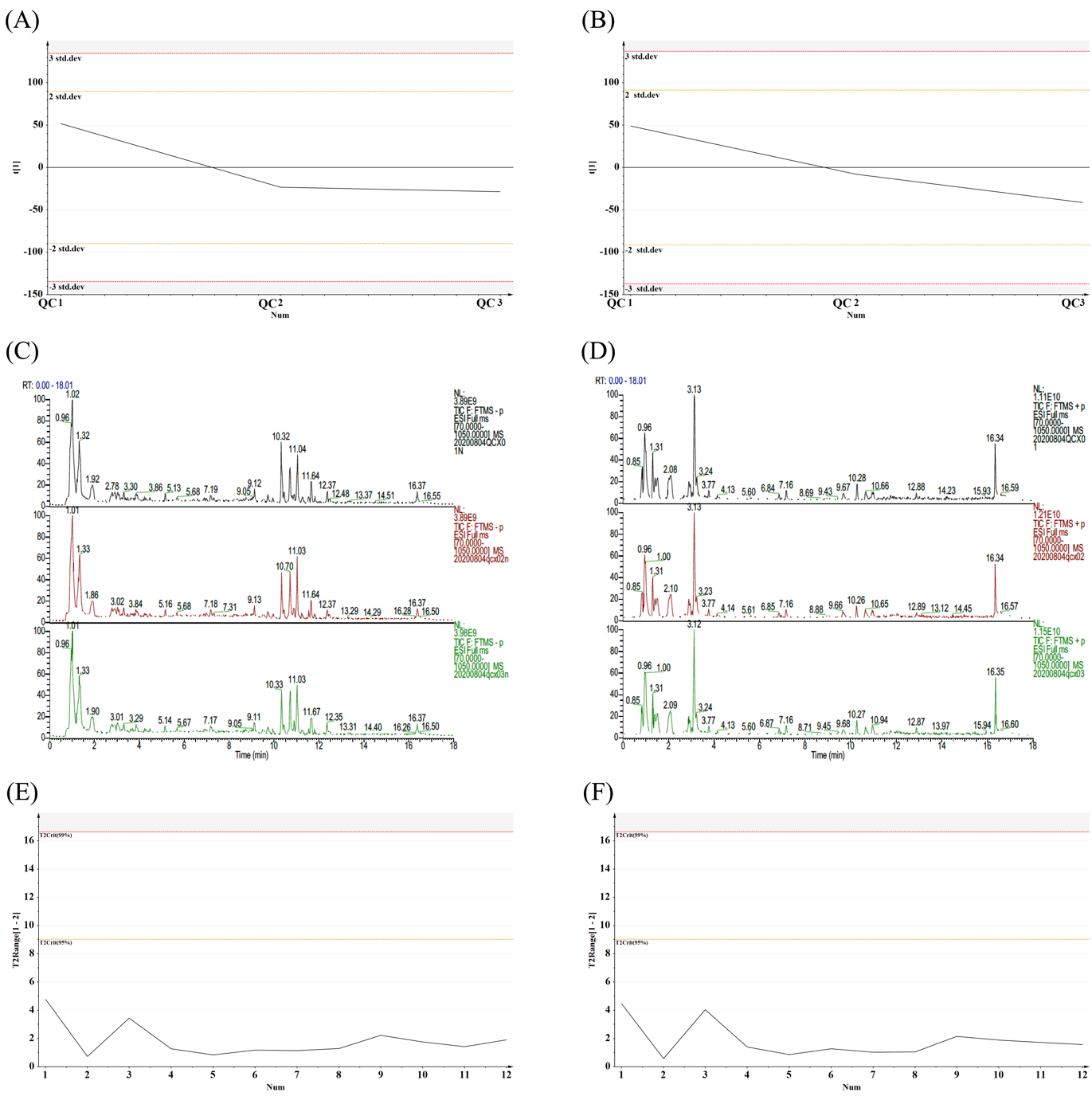


**Supplementary Figure 1.** Visual demonstration of the stability of the LC-MS/MS system. (**A**, **B**) The PCA score plots of the QC samples in ESI- and ESI+ modes, respectively; the X axis indicates the number of QC samples, and the Y axis indicates the range of RSD. (**C, D**) The base peak intensity (BPI) chromatograms of the QC samples in ESI- and ESI+ modes, respectively. (**E, F**) The line plot of the normalized QC samples in ESI- and ESI+ modes, respectively; the X axis indicates the number of QC samples, and the Y axis indicates the 95% confidence interval.





**Supplementary Figure 2.** The additional results of multivariate data analysis. The CV scores plots (**A**, **B**) of the samples in ESI- and ESI+ modes, respectively; the F factor and *p-*value of CV-ANOVA analysis assessing the reliability of the PLS-DA analysis are indicated in the figures. The Permutation plots for L3 variable (**C, D**) in ESI- and ESI+ modes, respectively; the correlation coefficient between the original y-variable and the permuted y-variable on the x-axis versus the cumulative *R^2^* and *Q^2^* on the Y-axis is shown. The intercept, as a measure of the overfit is indicated in the figures.

# Supplementary Tables

**Supplementary Table 1.** List of peak intensities identified in the study in negative ionization mode (ESI-).

**Supplementary Table 2.** List of peak intensities identified in the study in positive ionization mode (ESI+).

**Supplementary Table 3.** List of peak intensities identified in the study after normalization against QC samples in negative ionization mode (ESI-).

**Supplementary Table 4.** List of peak intensities identified in the study after normalization against QC samples in positive ionization mode (ESI+).

**Supplementary Table 5.** List of metabolites identified in the study in negative ionization mode (ESI-).

**Supplementary Table 6.** List of metabolites identified in the study in positive ionization mode (ESI+).

**Supplementary Table 7.** List of common and unique metabolites identified in the study between negative and positives ionization modes (ESI-/+).

**Supplementary Table 8.** List of statistically significantly different metabolites between L3 and L4 *A. simplex* developmental stages identified in the study in negative ionization mode (ESI-).

**Supplementary Table 9.** List of statistically significantly different metabolites between L3 and L4 *A. simplex* developmental stages identified in the study in positive ionization mode (ESI+).

**Supplementary Table 10.** List of common and unique metabolites for each of *A. simplex* developmental stage (L3 and L4) identified in the study in negative and positives ionization modes and (ESI-/+).

**Supplementary Table 11.** Assignment of metabolites identified in the study in negative ionization mode (ESI-) to main compound groups.

**Supplementary Table 12.** Assignment of metabolites identified in the study in positive ionization mode (ESI+) to main compound groups.

**Supplementary Table 13.** Pathways enrichment analysis of metabolites identified in the study in negative ionization mode (ESI-).

**Supplementary Table 14.** Pathways enrichment analysis of metabolites identified in the study in positive ionization mode (ESI+).
